# Inequality in early childhood neurodevelopment in six poor rural counties of China: a decomposition analysis

**DOI:** 10.1186/s12939-017-0691-y

**Published:** 2017-12-08

**Authors:** Cuihong Zhang, Chunxia Zhao, Xiangyu Liu, Qianwei Wei, Shusheng Luo, Sufang Guo, Jingxu Zhang, Xiaoli Wang, Robert W. Scherpbier

**Affiliations:** 1 Department of Child, Adolescent and Women’s Health, School of Public Health, Peking University, Beijing, China; 2 UNICEF Office for China, Beijing, China; 3 Department of Biostatistics, School of Public Health, The University of Texas Health Science Center at Houston, Houston, USA

**Keywords:** Early childhood neurodevelopment, Age and stages questionnaire, Health inequality, Poor rural China

## Abstract

**Background:**

Previous studies about inequality in children’s health focused more on physical health than the neurodevelopment. In this study, we aimed to evaluate the inequality in early childhood neurodevelopment in poor rural China and explore the contributions of socioeconomic factors to the inequality.

**Method:**

Information of 2120 children aged 0 to 35 months and their households in six poor rural counties of China was collected during July – September, 2013. Age and Stages Questionnaire-Chinese version, concentration index and decomposition analysis were used to assess the neurodevelopment of early childhood, measure its inequality and evaluate the contributions of socioeconomic factors to the inequality, respectively.

**Result:**

The prevalence of suspected developmental delay in children under 35 months of age in six poor rural counties of China was nearly 40%, with the concentration index of −0.0877. Household economic status, caregivers’ depressive symptoms, learning material and family support for learning were significantly associated with children’s suspected developmental delay, and explained 34.1, 14.1, 8.9 and 7.0% of the inequality in early childhood neurodevelopment, respectively.

**Conclusion:**

The early childhood neurodevelopment in the surveyed area is poor and unfair. Factors including household economic status, caregivers’ depressive symptoms, learning material and family support for learning are significantly associated with children’s suspected developmental delay and early developmental inequality. The results highlight the urgent need of monitoring child neurodevelopment in poor rural areas. Interventions targeting the caregivers’ depressive symptoms, providing learning material and developmental appropriate stimulating activities may help improve early childhood neurodevelopment and reduce its inequality.

**Electronic supplementary material:**

The online version of this article (doi:10.1186/s12939-017-0691-y) contains supplementary material, which is available to authorized users.

## Background

Early childhood, marked by the most rapid development and the strongest plasticity, especially in the central nervous system, is the most crucial phrase throughout the lifespan [1]. Early childhood neurodevelopment includes the development in language, motor, problem solving and social-emotional domains, etc. The term developmental delay is frequently used to identify children with delay in one or more above domains [2]. Previous studies have shown that children with developmental delay would have higher risks of poor school performance, career development and mental health in the future [3–6]. Furthermore, the poor development in early life may also bring heavy psychological burden to their family [7]. For instance, Webster et al. [8] found that 42% of the parents of children with developmental delay had a clinically significant parenting stress. Cheng et al. [9] reported that 26.9% of mothers of children with cognitive delay had depressive symptoms. Nonetheless, previous experimental studies have also shown that children could better achieve their developmental potential if given adequate nutrition, cognitive stimulations and learning opportunities during their early childhood [10–13]. These studies provide a chance to improve developmental outcomes in early childhood.

It’s estimated that about 17 million children under 5 years of age in China failed to achieve their full developmental potential [14, 15]. Due to the poor sanitation, deficient healthcare, low maternal education, increased maternal depression and inadequate early stimulations, children in poor rural areas are more vulnerable to suppressed developmental potentials [14]. Moreover, compared with caregivers in urban areas, caregivers in poor rural areas may care less about their children’s neurodevelopment, resulting in late detection and interventions of children with developmental delay.

Health inequality is unnecessary and unfair difference in health observed in populations [16]. And it is an ethical imperative to ensure all people attain their health, irrespective of their social-economic status, race, gender, etc. [17]. Averages health status cannot provide a complete representation of changes in health in a population and it may conceal the true disparity among different populations [18]. So the reduction of health inequality is another important goal beyond the improvement of average health status.

Early childhood is a period that can exert determining influence on subsequent life. Therefore, promoting children’s health and reducing their health inequality becomes an imperative for all national and international communities. Studies focusing on inequality in children’s health show that there are some inequalities existing in children’s physical health, and that the main contributing factors of inequality in children’s physical health are living conditions, household economic status and maternal education [19–24]. However, study of inequality in children’s neurodevelopment are still limited [25], especially in China. So we conducted this study to evaluate early childhood neurodevelopment and its inequality in poor rural China, and to explore the contributions of socioeconomic factors to the inequality.

## Methods

### Study subjects

The data used in this study came from the baseline investigation of Integrated Early Childhood Development Project, a community-based intervention program, funded by United Nations International Children’s Emergency Fund (UNICEF). This baseline investigation was conducted from July, 2013 to September, 2013. Given the differences in culture, parenting behaviors and ethnic groups between North and South China, Shanxi Province in North China and Guizhou Province in South China were selected. Three counties in each province were randomly selected from the list of nationally designed concentrated poverty battlefields by National Health and Family Planning Commission (NHFPC) and UNICEF. A total of 83 villages were further selected from a total of 856 villages meeting the following criteria: 1) the number of children under 3 years of age should be more than 50 (10% of the villages could have less than 50 children), 2) the villages can be reached by motor vehicles, 3) the township should have health center and maternal and child health staffs. The average population size of these 83 villages in 2012 was 1719.6 ± 774.7. And the median of per capita income of these villages was 1500 Chinese Yuan (CNY) (inter-quartile range (IQR) 760 ~ 2300 CNY). All the households with children aged 0 to 35 months in these 83 villages were contacted by phone based on the information of children’s healthcare management system and informed to bring the children to the primary healthcare institution for investigation. According to the information of children’s healthcare management system, there were 4288 children aged 0 to 35 months in these 83 villages. But some children failed to participate in the investigation due to the migration and the inconvenient traffic in remote mountainous areas. Finally, 2953 children were surveyed, accounting for 68.9% of children registered in these areas. Among the 2953 children surveyed, 835 were excluded for missing information (183 for missing height or weight, 75 for missing developmental score, 575 for missing household income), leaving a total of 2120 children in this analysis.

### Study instruments

#### Questionnaire

Face-to-face interviews were conducted with children’s caregivers by trained local healthcare providers using a standard structured questionnaire, which includes questions about the households, caregivers and children. All of the questions were derived from questionnaires of UNICEF’s 5th Multiple Indicator Cluster Survey (MICS5) [26].

Household per capita net income was used to reflect household economic status. Several questions about household income and cost of production were asked. The former includes household labor income, business income, transfer income and asset income. The latter is defined as the cost incurred to earn the income, including transport fee of migrant workers, material cost of business, and cost of seeds, fertilizers and pesticides for agricultural production, etc. Household net income was calculated as the difference between total income and total cost of production. Then household per capita net income was calculated as household net income / number of household members. Learning material and family support for learning were used to reflect the resources and stimulations children obtained. Children with picture books or toys were considered as having learning material. And according to the indicator list accompanying the questionnaires of MICS5 [26], children engaged four or more of the following activities with household members within 3 days before the survey were considered as having family support for learning: 1) reading books to the child, 2) telling stories to the child, 3) singing songs to or with the child, 4) taking child outside the home, 5) playing games with the child, 6) naming or counting things to or with the child.

#### Zung Self-rating Depression Scale (ZSDS)

ZSDS was used to assess the depressive symptoms of the caregivers. ZSDS is a 20-item self-reported questionnaire that is widely used as a screening tool, covering affective, psychological and somatic symptoms associated with depression [27]. There are four options for each item - a little of the time, some of the time, good part of the time, most of the time, and the corresponding responses are scored 1, 2, 3, 4 for the positively worded items and 4, 3, 2, 1 for the negatively worded items. An overall index score for the ZSDS is computed by summing each item’s score and dividing the sum by 0.8, resulting in a range of 25 to 100. Caregivers with index scores of 50 or more were identified as depression [28].

#### World Health Organization (WHO) child growth standards

Children’s height and weight were measured by two trained investigators during the study. The Z-scores of height-for-age and weight-for-height, calculated according to the WHO child growth standards published in 2006 [29], were used to evaluate children’s growth. Height-for-age Z-score less than −2 was defined as stunting, and weight-for-height Z-score less than −2 was defined as wasting.

#### Age and Stages Questionnaire (ASQ)

The ASQ-Chinese version was used to assess the early childhood neurodevelopment. The ASQ is a parent-completed developmental screening instrument [30]. The ASQ-third edition, which consists of 21 age-specific questionnaires intended for children ages 1–66 months, was published in 2009 and introduced to China later [31–33]. Each questionnaire consists of 30 items, covering five domains: communication, gross motor, fine motor, problem solving, and personal-social. Parents were asked to evaluate their children’s ability on every item, and their responses of “yes”, “sometimes” and “not yet” were scored 10, 5 and 0 points, respectively. The score of each domain was the sum of its corresponding six items’ scores. In this study, children were defined as suspected developmental delay (SDD) if the scores of one or more domains fell below the U.S. cut-off values.

All the variables used in this study are summarized in Table 1.

**Table 1:** Summary of all the variables in this study

Variable	Study instrument (Definition)	Type of variable
Household		
Region	Questionnaire	Categorical (Shanxi, Guizhou)
Single-child family	Questionnaire	Categorical (Yes, No)
Per capita net income	Questionnaire $\left(\frac{\mathrm{Total}\ \mathrm{income}-\mathrm{Total}\ \mathrm{cost}\ \mathrm{of}\ \mathrm{production}}{\mathrm{Number}\ \mathrm{of}\ \mathrm{household}\ \mathrm{members}}\right)$	Continuous (CNY)
Caregiver		
Age	Questionnaire	Continuous (Year)
Gender	Questionnaire	Categorical (Male, Female)
Role	Questionnaire	Categorical (Mother, Not mother)
Education	Questionnaire	Categorical (Illiterate, Primary school, Middle school, High school or above)
Depression	Zung Self-rating Depression Scale (Scores >50)	Categorical (Yes, No)
Child		
Age	Questionnaire	Continuous (Month)
Gender	Questionnaire	Categorical (Male, Female)
Stunting	WHO Child Growth Standards (Height-for-age Z-score < −2)	Categorical (Yes, No)
Wasting	WHO Child Growth Standards (Weight-for-height Z-score < −2)	Categorical (Yes, No)
Learning material	Questionnaire (Have picture books or toys)	Categorical (Yes, No)
Support for learning	Questionnaire (Engaged four or more activities)	Categorical (Yes, No)
Developmental delay	Age and Stages Questionnaire (Scores of one or more domains fall below the U.S. cut-off points)	Categorical (Yes, No)

### Statistical analysis

The prevalence and the concentration index (C) of SDD were used to reflect the health status of children’s neurodevelopment and its inequality. Univariate and multivariate logistic regression analyses and odds ratio (OR) were used to assess the associations between socioeconomic factors and early childhood neurodevelopment. Univariate analyses were conducted first, and variables with *p*-value <0.2 were selected for the multivariate analyses. Decomposition analysis of concentration index was used to assess the contributions of socioeconomic factors to the inequality in early childhood neurodevelopment. In addition, we conducted two sensitivity analyses to test the robustness of our primary analyses. First, we imputed the missing income by the average of per capita income of their corresponding villages to test the impact of missing data. Second, we identified children with SDD by using cutoff points stemmed from our own data (10th and 15th percentile) to test the impact of different definitions of children’s SDD. Then the main analyses were repeated, including the concentration index of children’s SDD, the associations between potential factors and children’s SDD, and the contributions of these factors to the inequality in children’s SDD.

**Fig. 1:**
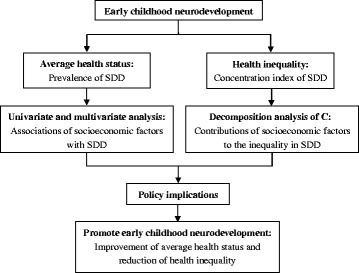
The framework of this study

Statistical significance was defined *P* < 0.05 (for 2-side tests). All statistical analyses were performed with STATA, version 12.0. Figure 1 shows the framework of analyses in this study.

#### Concentration index

The concentration index, which ranges from −1 to 1, provides a measure of the extent of inequality in health that is associated with economic status [34]. The index can be calculated by the following equation:

1 $$ C=\frac{2}{\mu}\mathit{\operatorname{cov}}\left({h}_i,{r}_i\right) $$

where *h*_*i*_ is the health status of the *i* ^th^ individual, *r*_*i*_ is the fractional rank of the *i* ^th^ individual in terms of household per capita net income and *μ* is the mean of the health status. A positive (negative) index suggests the health variable concentrated among the rich (poor). A value of zero suggests there is no inequality and a value of 0.2 to 0.3 is considered to be a high level of inequality, according to *Health Inequality Monitoring*, a book published by WHO [35].

### Decomposition analysis of concentration index

The decomposition analysis of concentration index allows us to estimate the contributions of socioeconomic factors to the inequality in health [22]. Wagstaff et al. showed that for any linear regression model linking a variable of interest, *y*, to a set of *k* determinants, *x*_*k*_:

2 $$ y=\alpha +\sum {\beta}_k{x}_k+\varepsilon $$

where *β*_*k*_ are the coefficients and *ε* is the error term. Given the relationship in Eq. (2), the concentration index for *y*, *C*, can be written as:

3 $$ C=\sum \left(\frac{\beta_k{\overline{x}}_k}{\mu}\right){C}_k+\frac{GC_{\varepsilon }}{\mu } $$

where *μ* is the mean of *y*, ${\overline{x}}_k$ is the mean of *x*_*k*_, and *C*_*k*_ is the concentration index for *x*_*k*_ (defined analogously to *C*). In the last term, *GC*_*ε*_ is a generalized concentration index for *ε*. The contribution rate (*CR*) of socioeconomic factor to inequality in *y*, can be written as:

4 $$ {CR}_{x_k}=\frac{\left({\beta}_k{\overline{x}}_k\right)/\mu }{C}{c}_k $$

For binary health variables, linear regression model like Eq. (2) is not appropriate. So marginal effects of covariates were calculated from binary logistic regression to replace *β*_*k*_ in the decomposition analysis [18].

## Results

### Demographic information

Table 2 shows the demographic characteristics of the households, caregivers and children. Among the 2120 households, the median of household per capita net income was 2000 CNY (IQR 666.7 ~ 5000.0 CNY), and 50.8% were below the poverty line in China (2300 CNY). For caregivers, the mean age was 29.5 ± 9.2 years, 80.7% were mothers, 39.0% had depressive symptoms and 68.3% completed 9-year compulsory education, but only 14.4% completed the 12-year education of senior high school. Among the 2120 children, the mean age was 18.6 ± 9.6 months, 56.8% were boys, 89.8% had learning material and 85.8% had family support for learning. The prevalence of stunting and wasting was 13.6 and 3.4%, respectively.

**Table 2:** Demographic characteristic of household, caregiver and child

	Factors	Value	N (%)
Household	Region	Shanxi	1057 (49.9)
		Guizhou	1063 (50.1)
	Single-child family	Yes	1005 (47.4)
		No	1115 (52.6)
	Per capita net income (CNY)	<2300	1076 (50.8)
		≥2300	1044 (49.2)
Caregiver	Age (year)	<20	41 (1.9)
		20–34	1728 (81.5)
		35–49	208 (9.8)
		≥50	143 (6.8)
	Gender	Male	322 (15.2)
		Female	1798 (84.8)
	Role	Mother	1711 (80.7)
		Not mother	409 (19.3)
	Education	Illiterate	179 (8.5)
		Primary School	492 (23.2)
		Middle School	1143 (53.9)
		High School or above	306 (14.4)
	Depression	Yes	826 (39.0)
		No	1294 (61.0)
Child	Age (month)	0–5	221 (10.4)
		6–11	383 (18.1)
		12–23	791 (37.3)
		24–35	725 (34.2)
	Gender	Male	1204 (56.8)
		Female	916 (43.2)
	Stunting	Yes	289 (13.6)
		No	1831 (86.4)
	Wasting	Yes	72 (3.4)
		No	2048 (96.6)
	Learning material	Yes	1904 (89.8)
		No	216 (10.2)
	Support for learning	Yes	1818 (85.8)
		No	302 (14.2)

### Prevalence and concentration index of SDD

Table 3 presents the prevalence and concentration index of SDD in children aged 0 to 35 months. The prevalence of SDD was 37.3%, and the prevalence of SDD in communication, gross motor, fine motor, problem solving and personal-social domain was 10.8, 17.6, 20.0, 17.5 and 17.5%, respectively. The concentration index of SDD in at least one of the domains was −0.0877, and the concentration indexes of SDD in communication, gross motor, fine motor, problem solving and personal-social domain were −0.1241, −0.1013, −0.1280, −0.1602 and −0.1304, respectively.

**Table 3:** Prevalence and concentration index of suspected developmental delay

Domain	Developmental delay N (%)	Concentration index (95% CI)
Communication	228 (10.8)	−0.1241 (−0.1941, −0.0541)
Gross motor	373 (17.6)	−0.1013 (−0.1540, −0.0486)
Fine motor	425 (20.0)	−0.1280 (−0.1761, −0.0800)
Problem solving	370 (17.5)	−0.1602 (−0.2137, −0.1066)
Personal-social	370 (17.5)	−0.1304 (−0.1824, −0.0784)
Overall developmental delay	791 (37.3)	−0.0877 (−0.1194, −0.0560)

### Associations of SDD with socioeconomic factors

Table 4 presents associations of SSD with household, caregiver and child factors. Ten variables were identified potentially associated with SDD according to the univariate analysis and then selected for the multivariate logistic regression analysis. The multivariate regression results show that household per capita net income (OR: 0.97; 95% CI: 0.95, 0.99), the depressive symptoms of caregivers (OR: 2.30; 95% CI: 1.89, 2.81), the age of children (OR: 0.94; 95% CI: 0.93, 0.95), children with stunting (OR: 1.32; 95% CI: 1.01, 1.74), learning material (OR: 0.53; 95% CI: 0.38, 0.73) and family support for learning (OR: 0.67; 95% CI: 0.51, 0.87) were significantly associated with SDD.

**Table 4:** Associations of socioeconomic factors with suspected developmental delay

	Developmental delay^a^		Developmental delay^b^	
	OR (95%CI)	*P*-value	OR (95%CI)	*P*-value
Household				
Region (Guizhou vs. Shanxi)	1.53 (1.28, 1.83)	<0.001	1.13 (0.92, 1.39)	0.241
Single-child family (Yes vs. No)	0.93 (0.78, 1.11)	0.419		
Per capita net income (1000 CNY)	0.96 (0.94, 0.98)	<0.001	0.97 (0.95, 0.99)	0.024
Caregiver				
Age (year)	1.01 (0.99, 1.02)	0.210		
Gender (Male vs. Female)	1.01 (0.79, 1.30)	0.920		
Role (Mother vs. Not mother)	0.96 (0.77, 1.20)	0.699		
Education (Middle school or above vs. Primary school or below)	0.80 (0.66, 0.96)	0.020	0.84 (0.67, 1.04)	0.105
Depression (Yes vs. No)	2.04 (1.71, 2.45)	<0.001	2.30 (1.89, 2.81)	<0.001
Child				
Age (month)	0.94 (0.93, 0.95)	<0.001	0.94 (0.93, 0.95)	<0.001
Gender (Male vs. Female)	1.20 (1.00, 1.43)	0.049	1.21 (1.00, 1.47)	0.053
Stunting (Yes vs. No)	1.25 (0.97, 1.61)	0.090	1.32 (1.01, 1.75)	0.048
Wasting (Yes vs. No)	1.81 (1.13, 2.91)	0.010	1.49 (0.90, 2.47)	0.121
Learning material (Yes vs. No)	0.30 (0.22, 0.40)	<0.001	0.53 (0.38, 0.73)	<0.001
Support for learning (Yes vs. No)	0.42 (0.33, 0.53)	<0.001	0.67 (0.51, 0.87)	0.003

^a^Univariate analysis; ^b^Multivariate analysis

### Contributions of socioeconomic factors to the inequality in SDD

The contributions of the socioeconomic factors to the inequality in SDD are presented in Table 5. The decomposition analysis of concentration index suggested that the ten factors included in the multivariate regression model explained 80.2% of the inequality in SDD, leading by household per capita net income (34.1%). Inequality in caregivers’ depressive symptoms, learning material and family support for learning explained 14.1, 8.9 and 7.0% of the inequality in SDD, respectively.

**Table 5:** Contributions of socioeconomic factors to inequality in suspected developmental delay

	Mean ${\overline{x}}_k$	Marginal effect *β*_*k*_	Elasticity ${\beta}_k{\overline{x}}_k/\mu$	Concentration index *c*_*k*_	Contribution $\left({\beta}_k{\overline{x}}_k/\mu \right){c}_k$	Contribution rate (%) $\frac{\left({\beta}_k{\overline{x}}_k\right)/\mu }{c}{c}_k\times 100\%$
Household						
Live in Guizhou	0.5014	0.0247	0.0332	−0.1302	−0.0043	4.9
Per capita net income (1000 CNY)	3.6730	−0.0054	−0.0532	0.5623	−0.0299	34.1
Caregiver						
Middle school or above	0.6835	−0.0366	−0.0670	0.0868	−0.0058	6.6
Depression	0.3896	0.1734	0.1811	−0.0682	−0.0123	14.1
Child						
Age (month)	18.5693	−0.0125	−0.6223	0.0011	−0.0007	0.8
Male	0.5679	0.0383	0.0583	−0.0082	−0.0005	0.5
Stunting	0.1363	0.0569	0.0208	−0.1062	−0.0022	2.5
Wasting	0.0340	0.0831	0.0076	−0.0747	−0.0006	0.6
Learning material	0.8981	−0.1364	−0.3284	0.0238	−0.0078	8.9
Support for learning	0.8575	−0.0844	−0.1941	0.0318	−0.0062	7.0

*u*, *c* represent the prevalence and concentration index of suspected developmental delay, respectively

### Sensitivity analyses

One sensitivity analysis was conducted by imputing data for missing income. The results are shown in Additional file 1: Tables S1–S3. Although the prevalence of SDD became slightly higher, the other main findings were still similar to the primary results, such as the medium-high level of inequality in children’ s SDD, the significant associations between household economic status, caregivers’ depressive symptoms, learning material, family support for learning and children’s SDD, and these factors’ main contributions to the inequality of children’s SDD.

Another sensitivity analysis was also done to test the impact of different definitions of children’ SDD. The results are shown in Additional file 1: Tables S4–S6. Almost all the main findings of the primary analyses were also confirmed in this sensitivity analysis.

## Discussion

Our study revealed a high prevalence of SDD (37.4%) in six poor rural counties of China. It was much higher than the result of another Chinese study (27.1%) conducted in southern urban in children aged 6 to 30 months using ASQ-Chinese version [36], and one study (8.3%) conducted in Turkish children aged 3 to 36 months using ASQ-Turkish Version [37]. It is generally estimated the prevalence of confirmed developmental delay was about 10 to 20%, with significantly higher rates among children living in poverty [38]. On one hand, the high prevalence of SDD in our study might be a product of an imperfect measurement. It might be overestimated by using a Chinese-adapted screening tool but U.S. cutoff points [39]. On the other hand, the high prevalence might also result from the disadvantaged factors in poor rural areas and indicate an urgent need for early detection and interventions for children at risks of SDD. Therefore, there is an urgent need to incorporate the developmental monitoring into the existing well-child healthcare visits at primary care level to ensure timely identification and early interventions for children with SDD.

The concentration index of SDD ranged from −0.0877 to −0.1602 in our study, suggesting the existence of medium-high level of inequality in SDD, which concentrated among the poor, according to *Health Inequality Monitoring* by WHO and the study of Wagstaff [35, 40]. A study conducted in Indonesian also showed similar results in children aged 7 to 14 years [25]. Further studies are needed since the evidence of inequality in early childhood neurodevelopment is still limited.

Although household economic status was weakly associated with children’s SDD, it was the most important factor in explaining the inequality in children’s SDD in our study. Family is a key nurturing environment for children. Its economic status will affect children’s living conditions and their access to resources of nutrition, education and health. Previous studies have shown that poverty would severely limit children’s developmental potential [1, 14, 41–43]. For instance, poor areas might not be able to provide nutritious food, poverty could limit the availability of educational resources and the utilization of healthcare services for children. Furthermore, inequality in socioeconomic resources could result in inequality in ECD [1]. WHO states that “Any additional gain in social and economic recourses to a given family could result in commensurate gains in the development of the children in that family” [1]. Therefore, we suggest the government provide social and economic support to poor families, especially those with young children. For instance, micronutrient supplementation, age appropriate picture books and toys could be provided for children of poor families.

In our study, we found caregivers’ depressive symptoms were strongly associated with children’s SDD and the second leading factor in explaining the inequality in SDD. As we know, children’s early contact with outside environment is mostly mediated and controlled by caregivers. The daily interactions between caregivers and children constitute a main source of children’s early stimulations. Previous studies have shown that caregivers with depression would neglect children’s demands for nutrition, and emotional and cognitive stimulations [44–47]. Moreover, the adverse effects of maternal depression on the development of children are independent of the effects of poverty, malnutrition and other social disadvantages [48]. Identifying caregivers with depressive symptoms and taking timely, effective interventions may be one of the strategies to improve children’s neurodevelopment and reduce the inequality [49]. Thus, we recommend that it’s a priority to screen caregivers’ depressive symptoms, and this could be done during well-child visits by primary healthcare providers using a standardized screening tool.

We found learning material and family support for learning were significantly associated with children’s SDD, and explained 8.9 and 7.0% of the inequality in SDD, respectively. Previous studies have shown that effective early stimulations could boost children’s neurodevelopment [10, 13, 50, 51]. Effective investments in ECD also have the potential to reduce inequalities perpetuated by poverty, poor health, ect [52]. So it is recommended to implement ECD programs in poor areas, providing age-appropriate picture books, early learning opportunities to children and specific recommendations to caregivers about play, communication and parent-child interaction to improve children’s developmental outcomes and diminish the inequalities.

Although we didn’t find a statistically significant association between caregivers’ education and children’s SDD, we still included this factor into the decomposition analysis of concentration index of children’s SDD because previous studies have shown that caregivers’ education is an important factor for children’s development [49, 53]. Previous studies have shown that caregivers with lower education might have less healthcare knowledge, make less use of healthcare services, pay less attention to children’s development and have less decision making power to allocate family resources to children than caregivers with higher education [54–57]. What’s more, caregivers with lower education would have higher risks of developing depression [58]. The neglect of children’s demands for cognitive stimulations and the decrease of parent-child interactions caused by caregiver’s depression might negatively influence early childhood neurodevelopment [45, 46]. So, caregivers’ education might affect children’s development through these mechanisms, and then influence its inequality. In order to reduce the inequality in education as well as the inequality in offspring’s early development, we suggest further expanding the coverage of 9-year compulsory education in poor rural China and providing free high school education for poor children preferentially.

Studies about inequality in early childhood neurodevelopment are still limited, especially in China, so our study is of great significance for filling the gap. This study not only provided evidence on the general information of early childhood neurodevelopment and its inequality in poor rural China, but also investigated the contributions of socioeconomic factors to the inequality in early childhood development through the decomposition analysis of concentration index. In addition, sensitivity analyses were conducted to test the robustness of the results. The findings from the primary analyses and the sensitivity analyses both confirmed that the neurodevelopment of early childhood in the surveyed area is poor and unfair, and that some factors are significantly associated with children’s SDD and its inequality. The results of this study has great implication to combat the large number of children with developmental risks in China and other countries.

There are several limitations in this study. First, it is a cross-sectional study, which only allows us to identify the associations but not causal relationships. Further studies such as community intervention trial are needed to establish the causal relationships. Second, the representative of the sample is limited. The 83 villages in six counties of two provinces were selected according to certain criteria. Moreover, only 68.9% of the children registered in these villages were surveyed. Therefore, children surveyed in this study might not represent the entire population aged 0 to 35 months in the six poor rural counties of China. And cautions must be taken to generalize our results to other populations or areas. Third, the prevalence of SDD may be overestimated by using the U.S. cutoff points. One study showed that children younger than 24 months in China norm group scored significantly lower than their peers in U.S. norm group [39]. However, the findings of this study were robust in the sensitivity analyses. That may confirm the validity of the results of this study. Fourth, we used self-reported income to represent the economic status of the surveyed households, which would bring some measurement errors into the study. But previous studies have shown that the measurement error from self-reported earnings isn’t a big cause of concern in practice [59]. In addition, in order to measure income accurately as much as possible, we revised the questions of income sources carefully after pre-investigation to adapt to local conditions. Fifth, some subjects failed to provide complete information, especially household income, which might introduce information bias to our results. But the main findings in the sensitivity analyses about missing data were similar to the results in the primary analyses. So excluding the missing data might be acceptable in this regard. Sixth, we used the number of activities children engaged with their household members within 3 days before the survey to represent the level of family support for learning, which might bring some measurement errors in this study. In the future, standard tools need to be developed. Last but not the least, other factors that may associate with children’s neurodevelopment such as children’s birth order, birth weight, iron deficiency anemia and domestic violence were not analyzed in this study. Further studies are needed to clarify these associations.

## Conclusion

The neurodevelopment of early childhood in the surveyed area is poor and unfair. The SDD was more prevalent in poor children. Factors including household economic status, caregivers’ depressive symptoms, learning material and family support for learning are significantly associated with children’s SDD and its inequality. The results highlight the urgent need of monitoring child neurodevelopment in poor rural areas. Interventions targeting the caregivers’ depressive symptoms, providing learning material and developmental appropriate stimulating activities may help improve early childhood neurodevelopment and reduce its inequality.

## Additional file

Additional file 1: Supplemental Tables Detailed information about sensitivity analyses. **Table S1.** Prevalence and concentration index of suspected developmental delay, with missing income imputed. **Table S2.** Associations of socioeconomic factors with suspected developmental delay, with missing income imputed. **Table S3.** Contributions of socioeconomic factors to inequality in suspected developmental delay, with missing income imputed. **Table S4.** Associations of socioeconomic factors with suspected developmental delay, with different cutoff points. **Table S5.** Concentration index of suspected developmental delay, with different cutoff points. **Table S6.** Contributions of socioeconomic factors to inequality in suspected developmental delay, with different cutoff points. (DOCX 25 kb)

## Abbreviations

ASQ: Age and Stages Questionnaire
C: Concentration index
CNY: Chinese Yuan
ECD: Early Childhood Development
IQR: Inter-quartile range
MICS5: 5th Multiple Indicator Cluster Survey
NHFPC: National Health and Family Planning Commission
OR: Odds ratio
SDD: Suspected developmental delay
UNICEF: United Nations International Children’s Emergency Fund
WHO: World Health Organization
ZSDS: The Zung Self-rating Depression Scale
