# Supplementary material for: Inequality in early childhood neurodevelopment in six poor rural counties of China: a decomposition analysis
Source: Int J Equity Health. 2017 Dec 8;16:212. doi: 10.1186/s12939-017-0691-y (PMC5723085; doi:10.1186/s12939-017-0691-y)
Supplement: Additional file 1: — Supplemental Tables Detailed information about sensitivity analyses. Table S1. Prevalence and concentration index of suspected developmental delay, with missing income imputed. Table S2. Associations of socioeconomic factors with suspected developmental delay, with missing income imputed. Table S3. Contributions of socioeconomic factors to inequality in suspected developmental delay, with missing income imputed. Table S4. Associations of socioeconomic factors with suspected developmental delay, with different cutoff points. Table S5. Concentration index of suspected developmental delay, with different cutoff points. Table S6. Contributions of socioeconomic factors to inequality in suspected developmental delay, with different cutoff points. (DOCX 25 kb) [file 12939_2017_691_MOESM1_ESM.docx]

**Additional file 1**

**Table S1.** Prevalence and concentration index of suspected developmental delay, with missing income imputed.

| Domain | Developmental delay N (%) | Concentration index (95% CI) |
| --- | --- | --- |
| Communication | 303 (11.2) | -0.1318 (-0.1921, -0.0716) |
| Gross motor | 489 (18.1) | -0.0965 (-0.1425, -0.0504) |
| Fine motor | 586 (21.7) | -0.1200 (-0.1600, -0.0801) |
| Problem solving | 505 (18.7) | -0.1527 (-0.1972, -0.1082) |
| Personal-social | 490 (18.2) | -0.1298 (-0.1747, -0.0850) |
| Overall developmental delay | 1061 (39.4) | -0.0803 (-0.1070, -0.0536) |

**Table S2.** Associations of socioeconomic factors with suspected developmental delay, with missing income imputed.

|  | Developmental delay | |  |
| --- | --- | --- | --- |
|  | OR (95%CI) | *P*-value |  |
| **Household** |  |  |  |
| Region (Guizhou vs. Shanxi) | 1.12 (0.94, 1.35) | 0.211 |  |
| Per capita net income (1000 CNY) | 0.97 (0.95, 0.99) | 0.008 |  |
| **Caregiver** |  |  |  |
| Education (Middle school or above vs. Primary school or below) | 0.85 (0.70, 1.02) | 0.080 |  |
| Depression (Yes vs. No) | 2.21 (1.86, 2.62) | ＜0.001 |  |
| **Child** |  |  |  |
| Age (month) | 0.94 (0.93, 0.95) | ＜0.001 |  |
| Gender (Male vs. Female) | 1.14 (0.96, 1.34) | 0.145 |  |
| Stunting (Yes vs. No) | 1.37 (1.08, 1.74) | 0.010 |  |
| Wasting (Yes vs. No) | 1.37 (0.89, 2.10) | 0.149 |  |
| Learning material (Yes vs. No) | 0.57 (0.44, 0.76) | ＜0.001 |  |
| Support-for-learning (Yes vs. No) | 0.59 (0.47, 0.73) | ＜0.001 |  |

**Table S3.** Contributions of socioeconomic factors to inequality in suspected developmental delay, with missing income imputed.

|  | Contribution | Contribution rate (%) |  |
| --- | --- | --- | --- |
| **Household** |  |  |  |
| Live in Guizhou | -0.0037 | 4.6 |  |
| Per capita net income (1000 CNY) | -0.0290 | 36.1 |  |
| **Caregiver** |  |  |  |
| Middle school or above | -0.0047 | 5.8 |  |
| Depression | -0.0126 | 15.7 |  |
| **Child** |  |  |  |
| Age (month) | 0.0003 | -0.4 |  |
| Male | -0.0004 | 0.5 |  |
| Stunting | -0.0021 | 2.6 |  |
| Wasting | -0.0006 | 0.8 |  |
| Learning material | -0.0066 | 8.3 |  |
| Support-for-learning | -0.0097 | 12.0 |  |

**Table S4.** Associations of socioeconomic factors with suspected developmental delay, with different cutoff points.

|  | 10% Cutoff points | |  | 15% Cutoff points | | |
| --- | --- | --- | --- | --- | --- | --- |
|  | OR (95%CI) | *P*-value |  | OR (95%CI) | *P*-value | |
| **Household** |  |  |  |  | |  |
| Region (Guizhou vs. Shanxi) | 1.05 (0.83, 1.33) | 0.675 |  | 1.07 (0.87, 1.32) | | 0.517 |
| Per capita net income (1000 CNY) | 0.97 (0.94, 1.00) | 0.040 |  | 0.96 (0.94, 0.99) | | 0.004 |
| **Caregiver** |  |  |  |  | |  |
| Education (Middle school or above vs. Primary school or below) | 0.78 (0.61, 0.99) | 0.043 |  | 0.80 (0.64, 0.99) | | 0.042 |
| Depression (Yes vs. No) | 2.96 (2.38, 3.69) | ＜0.001 |  | 2.55 (2.10, 3.09) | | ＜0.001 |
| **Child** |  |  |  |  | |  |
| Age (month) | 1.01 (0.99, 1.02) | 0.409 |  | 1.00 (0.99, 1.01) | | 0.740 |
| Gender (Male vs. Female) | 1.01 (0.81, 1.25) | 0.961 |  | 1.11 (0.92, 1.36) | | 0.275 |
| Stunting (Yes vs. No) | 1.56 (1.16, 2.09) | 0.003 |  | 1.45 (1.10, 1.90) | | 0.007 |
| Wasting (Yes vs. No) | 1.35 (0.76, 2.30) | 0.289 |  | 1.65 (0.99, 2.72) | | 0.051 |
| Learning material (Yes vs. No) | 0.49 (0.35, 0.70) | ＜0.001 |  | 0.57 (0.41, 0.79) | | 0.001 |
| Support-for-learning (Yes vs. No) | 0.74 (0.55, 1.00) | 0.044 |  | 0.75 (0.57, 0.98) | | 0.037 |

**Table S5.** Concentration index of suspected developmental delay, with different cutoff points.

|  | Concentration index (95% CI) | | | |
| --- | --- | --- | --- | --- |
|  | | 10% Cutoff points | 15% Cutoff points | |
| Communication | | -0.0903 (-0.1696, -0.0111) | | -0.0936 (-0.1567, -0.0306) |
| Gross motor | | -0.1299 (-0.2079, -0.0519) | | -0.1026 (-0.1658, -0.0394) |
| Fine motor | | -0.2417 (-0.3209, -0.1626) | | -0.2034 (-0.2662, -0.1406) |
| Problem solving | | -0.2173 (-0.3008, -0.1338) | | -0.2009 (-0.2621, -0.1396) |
| Personal-social | | -0.1357 (-0.2170, -0.0545) | | -0.1452 (-0.2067, -0.0837) |
| Overall developmental delay | | -0.1342 (-0.1813, -0.0872) | | -0.1100 (-0.1465, -0.0733) |

**Table S6.** Contributions of socioeconomic factors to inequality in suspected developmental delay, with different cutoff points.

|  | 10% Cutoff points | |  | 15% Cutoff points | |
| --- | --- | --- | --- | --- | --- |
|  | Contribution | Contribution rate (%) |  | Contribution | Contribution rate (%) |
| **Household** |  |  |  |  |  |
| Live in Guizhou | -0.0024 | 1.8 |  | -0.0029 | 2.6 |
| Per capita net income (1000 CNY) | -0.0429 | 31.9 |  | -0.0476 | 43.3 |
| **Caregiver** |  |  |  |  |  |
| Middle school or above | -0.0109 | 8.1 |  | -0.0087 | 7.9 |
| Depression | -0.0223 | 16.6 |  | -0.0168 | 15.3 |
| **Child** |  |  |  |  |  |
| Age (month) | 0.0001 | -0.1 |  | 0.0001 | -0.1 |
| Male | -0.0001 | 0.0 |  | -0.0003 | 0.3 |
| Stunting | -0.0050 | 3.7 |  | -0.0036 | 3.3 |
| Wasting | -0.0006 | 0.4 |  | -0.0009 | 0.8 |
| Learning material | 0.0238 | 9.2 |  | -0.0081 | 7.4 |
| Support-for-learning | -0.0062 | 4.6 |  | -0.0052 | 4.7 |
